# X‐Ray Activatable Au/Ag Nanorods for Tumor Radioimmunotherapy Sensitization and Monitoring of the Therapeutic Response Using NIR‐II Photoacoustic Imaging

**DOI:** 10.1002/advs.202206979

**Published:** 2023-02-15

**Authors:** Si Zheng, Duyang Gao, Yayun Wu, Dehong Hu, Ziyue Li, Yuenan Wang, Hairong Zheng, Yingjia Li, Zonghai Sheng

**Affiliations:** ^1^ Department of Medicine Ultrasonics Nanfang Hospital Southern Medical University Guangzhou 510515 P. R. China; ^2^ Paul C. Lauterbur Research Center for Biomedical Imaging Institute of Biomedical and Health Engineering Shenzhen Institute of Advanced Technology Chinese Academy of Sciences Shenzhen 518055 P. R. China; ^3^ Department of Radiation Oncology Peking University Shenzhen Hospital Shenzhen 518036 P. R. China

**Keywords:** activatable imaging, Au/Ag nanorods, photoacoustic imaging, radioimmunotherapy, second near‐infrared region

## Abstract

Radioimmunotherapy (RIT) is an advanced physical therapy used to kill primary cancer cells and inhibit the growth of distant metastatic cancer cells. However, challenges remain because RIT generally has low efficacy and serious side effects, and its effects are difficult to monitor in vivo. This work reports that Au/Ag nanorods (NRs) enhance the effectiveness of RIT against cancer while allowing the therapeutic response to be monitored using activatable photoacoustic (PA) imaging in the second near‐infrared region (NIR‐II, 1000–1700 nm). The Au/Ag NRs can be etched using high‐energy X‐ray to release silver ions (Ag^+^), which promotes dendritic cell (DC) maturation, enhances T‐cell activation and infiltration, and effectively inhibits primary and distant metastatic tumor growth. The survival time of metastatic tumor‐bearing mice treated with Au/Ag NR‐enhanced RIT is 39 days compared with 23 days in the PBS control group. Furthermore, the surface plasmon absorption intensity at 1040 nm increases fourfold after Ag^+^ are released from the Au/Ag NRs, allowing X‐ray activatable NIR‐II PA imaging to monitor the RIT response with a high signal‐to‐background ratio of 24.4. Au/Ag NR‐based RIT has minimal side effects and shows great promise for precise cancer RIT.

## Graphical abstract

This work reports X‐ray activatable Au/Ag core/shell nanorods (NRs) for tumor radioimmunotherapy (RIT) enhancement while achieving second near‐infrared region (NIR‐II) photoacoustic (PA) imaging to monitor the therapeutic response.

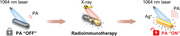

## 1 Introduction

Radiotherapy (RT) is an important physical strategy for cancer treatment,^[^ ^1^ ^]^ showing a good inhibitory effect on primary tumors.^[^ ^2^ ^]^ Moreover, recent studies have shown that local RT enables the release of some tumor‐associated antigens, initiates a systemic immune response, and has anticancer effects on distant metastatic tumors.^[^ ^3^ ^]^ Although RT has been effective in most animal antitumor experiments, further clinical application of RT is still challenging due to its inefficient immunogenic cell death (ICD) efficacy.^[^ ^4^ ^]^ To address this limitation, some studies have used a combination of RT and immunotherapy, named radioimmunotherapy (RIT), to improve the ICD efficacy.^[^ ^5^ ^]^ However, side effects and issues with patient tolerance limit the application of RIT.^[^ ^5^, ^6^ ^]^ Thus, it is extremely significant to develop a RIT approach with minimal side effects and high efficiency in inhibiting metastasis.

In recent years, multifunctional nanomedicines have been developed to deliver different immune agents, including immune checkpoint inhibitors,^[^ ^7^ ^]^ small‐molecule inhibitors,^[^ ^8^ ^]^ and adjuvants^[^ ^9^ ^]^ into the tumor region for enhanced RIT. These nanomedicines are generally composed of polymer carriers and immune reagents, among other components, and they exhibit high therapeutic efficacy for inhibiting metastatic tumor growth. Liu et al.^[^ ^10^ ^]^ reported polymer nanomedicines to encapsulate imiquimod (an immunoadjuvant) and catalase to modulate the immune‐suppressed microenvironment and enhance the therapeutic efficacy of RIT. Moreover, Gong et al.^[^ ^11^ ^]^ used multifunctional nanomedicines composed of polylysine, iron oxide, and CpG to enhance the in situ vaccination effect of RIT. Despite important advances, challenges remain due to the complex ingredients in nanomedicines and the difficulty in monitoring the response to RIT in vivo.

**Scheme 1:**
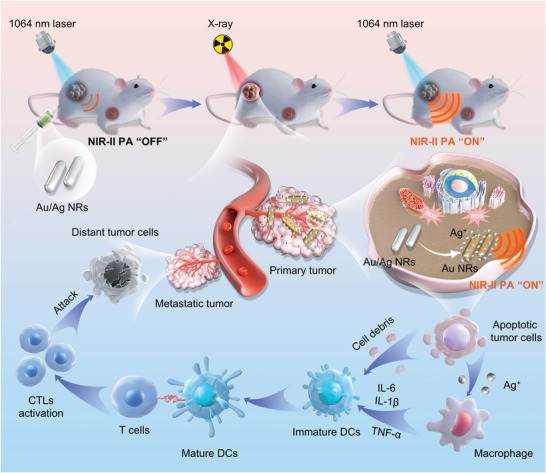
Schematic of Au/Ag nanorods (NRs) for tumor radioimmunotherapy (RIT) and X‐ray activated second near‐infrared region (NIR‐II) photoacoustic (PA) imaging. Au/Ag NRs were injected into tumor tissues intratumorally and irradiated using high‐energy X‐ray to produce reactive oxygen species (ROS) to kill cancer cells while releasing silver ions (Ag^+^) to trigger NIR‐II PA enhancement. Ag^+^ release stimulated macrophages to secrete cytokines tumor necrosis factor (TNF) *α*, interleukin 6 (IL‐6), and interleukin 1*β* (IL‐1*β*), promoting the maturation of dendritic cells (DCs) and tumor antigens and enhancing the activity of T lymphocytes to kill distant metastatic cancer cells.

Herein, we report the application of X‐ray activated Au/Ag core/shell nanorods (NRs) as radiosensitizing nanomedicines to enhance tumor RIT and monitor its therapeutic response using photoacoustic (PA) imaging in the second near‐infrared region (NIR‐II, 1000–1700 nm) (**Scheme** **1**). Au/Ag NRs can be etched by X‐ray to release silver ions (Ag^+^), in turn promoting dendritic cell (DC) maturation and enhancing the capability of T lymphocytes to kill cancer cells. Meanwhile, the release of Ag^+^ triggers a red shift of the surface plasmon absorption peak from the first near‐infrared region (NIR‐I, 650–950 nm) to the NIR‐II region. This allowed us to monitor the therapeutic response to RIT using activatable NIR‐II PA imaging in vivo with high sensitivity and large depth.^[^ ^12^ ^]^ The enhanced efficacy of RIT with the use of Au/Ag NRs was realized both in vitro and in vivo primary and metastatic tumor mouse models. The side effects of Au/Ag NR‐sensitized RIT were also evaluated by histological analysis, hematology, and biochemical assays. Our study provides a paradigm for single inorganic nanomedicine‐enhanced cancer RIT and in vivo imaging to monitor the response to RIT.

## 2 Results and Discussion

### 2.1 Characterization of X‐Ray Activatable Au/Ag NRs

**Figure 1:**
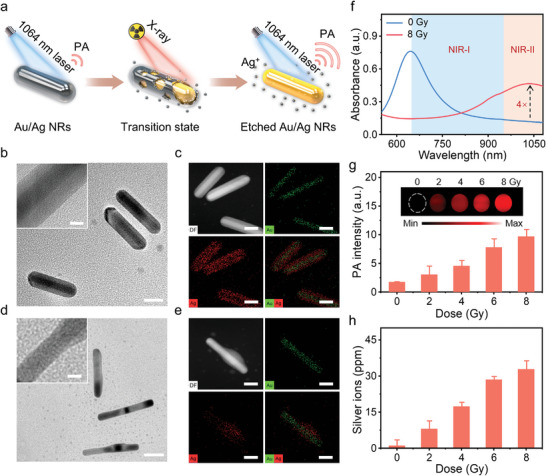
Characterization of X‐ray activated Au/Ag nanorods (NRs). a) Schematic of X‐ray activated Au/Ag NRs for release of silver ions (Ag^+^) and amplification of second near‐infrared region (NIR‐II) photoacoustic (PA) signals. b) Transmission electron microscopy (TEM) images of Au/Ag NRs. Scale bar: 20 nm. c) High‐angle annular dark‐field (HAADF) images and element mapping images of Au/Ag NRs. Scale bar: 10 nm. d) TEM images of Au/Ag NRs irradiated with X‐ray. Insets show high‐resolution TEM images of the corresponding NRs. X‐ray dose: 8 Gy; scale bar: 20 nm. e) HAADF images and element mapping images of Au/Ag NRs irradiated with X‐ray. X‐ray dose: 8 Gy; scale bar: 10 nm. f) Absorption spectra of Au/Ag NRs with or without X‐ray. X‐ray dose: 8 Gy. Time: 72 h. g) NIR‐II PA images of Au/Ag NRs irradiated with X‐ray. h) Quantitative analysis of released Ag^+^ from Au/Ag NRs irradiated with X‐ray.

Ag^+^ are released from Au/Ag NRs in response to high‐energy X‐ray irradiation,^[^ ^12^, ^13^ ^]^ leading to NIR‐II PA signal enhancement (**Figure** **1**a). To verify this hypothesis, Au/Ag NRs were prepared using the seedless growth method described previously (Figures S1–S3, Supporting Information).^[^ ^13^ ^]^ The transmission electron microscopy (TEM) and elemental mapping images revealed that the NRs had a representative core/shell nanostructure containing an Au core and an Ag shell (Figure 1b,c). The structure and elements of the NRs were further characterized under X‐ray (Dose: 8 Gy). The TEM and elemental mapping images demonstrate that most of the Ag nanolayer disappeared after X‐ray irradiation (Figure 1d,e; Figure S4, Supporting Information), resulting in an increase in the length/diameter ratio of the etched NRs from 3.86 ± 0.06 to 4.43 ± 0.09 (Figure S5, Supporting Information). The maximum surface plasmon absorption band red‐shifted from 700 to 1040 nm, and the absorbance at 1040 nm increased from 0.1 to 0.4 (Figure 1f). The increased absorption in the NIR‐II region of the etched NRs illustrates their good NIR‐II PA imaging ability. The PA signals of the NRs were gradually enhanced upon X‐ray irradiation of 0–8 Gy (Figure 1g). NIR‐II PA signal intensity of the NRs irradiated with an 8 Gy dose of X‐ray was 5.6‐times greater than that of the NRs treated without X‐ray irradiation, showing efficiently activated performance. Correspondingly, released Ag^+^ from the NRs increased with the irradiation dose, and the amount of Ag^+^ release reached 32.8 ppm at an X‐ray irradiation dose of 8 Gy (Figure 1h). Therefore, Au/Ag NRs could be activated by high‐energy X‐ray to release Ag^+^, which in turn enhanced NIR‐II PA imaging.

### 2.2 Effects of Hydrogen Peroxide (H_2_O_2_) and Hydrogen Ions (H^+^) on the X‐Ray Activated Performance of Au/Ag NRs

**Figure 2:**
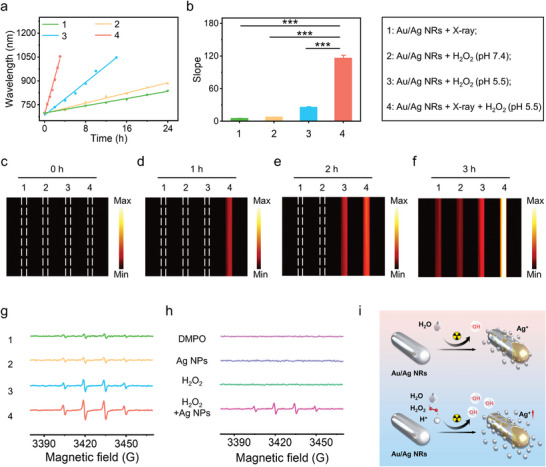
Effect of H_2_O_2_ and H^+^ on the X‐ray activated performance of Au/Ag nanorods (NRs). a) Linear relationship between the maximal absorption wavelength of the Au/Ag NRs and the reaction time. X‐ray dose: 8 Gy; *C*_Au/Ag NRs_: 50 µg mL^−1^; *C*_H2O2_: 5 × 10^−3^ m. b) Quantification of the line curve slopes of reactions 1–4 in a). ^*^ *p* < 0.05, ^**^ *p* < 0.01, ^***^ *p* < 0.001. c–f) Second near‐infrared region (NIR‐II) photoacoustic (PA) images of Au/Ag NRs under reactions 1–4 at different reaction times. g) Electron spin resonance (ESR) spectra of the Au/Ag NRs in reactions 1–4. X‐ray dose: 8 Gy; *C*_Au/Ag NRs_: 50 µg mL^−1^; *C*_H2O2_: 5 × 10^−3^ m. h) ESR spectra of dimethyl pyridine N‐oxide (DMPO), Ag nanoparticles (NPs), H_2_O_2_, and Ag NPs + H_2_O_2_. *C*_Ag NPs_: 50 µg mL^−1^; *C*_H2O2_: 5 × 10^−3^ m. i) Schematic of the reaction mechanism of Au/Ag NRs X‐ray etching.

Weak acidity (pH = 6.5–7.0) and abnormal H_2_O_2_ content (100 × 10^−6^ m to 1.0 × 10^−3^ m) are two characteristics of the tumor microenvironment.^[^ ^14^ ^]^ Au/Ag NRs are thought to react with H_2_O_2_ and H^+^ ions, accelerating the etching reaction rate. To verify this hypothesis, we investigated the effects of H_2_O_2_ (5 × 10^−3^ m) and weak acidity (pH = 5.5) on the absorption band of the Au/Ag NRs subjected to X‐ray irradiation (Figure S6, Supporting Information). The wavelengths of maximal absorption of Au/Ag NRs in reactions 1–4 red‐shifted toward the long wavelength over time, showing a good linear relationship (**Figure** **2**a). The etching reaction rate of reaction 4 was 116.3 nm h^−1^, which equated to a 20.4‐fold, 14.5‐fold, and 4.5‐fold increase compared with the reaction rates of reaction 1 (5.7 nm h^−1^), reaction 2 (8.0 nm h^−1^), and reaction 3 (25.9 nm h^−1^) (Figure 2b). The increased absorption intensity of Au/Ag NRs in the NIR‐II region was conducive to enhancing their NIR‐II PA imaging ability. Next, we measured the NIR‐II PA signals generated in reactions 1–4 over time using a commercial PA imaging system (Figure 2c–f). After 3 h of etching, the NIR‐II PA signal of reaction 4 was 117.4, which was 5.0‐times, 2.3‐times, and 1.4‐times greater than that of reactions 1, 2, and 3, respectively (Figure S7, Supporting Information), suggesting that H_2_O_2_ and H^+^ accelerated the activation rate of Au/Ag NRs irradiated with X‐ray, leading to a stronger NIR‐II PA signal.

To explore the possible etching reaction mechanism, electron spin resonance (ESR) spectra were assessed to detect the generation of hydroxyl radicals (·OH) (Figure 2g,h). X‐ray combined with Au/Ag NRs only produced a few ·OH (Figure 2g, reaction 1). In contrast, the abundance of ·OH significantly increased in the presence of H_2_O_2_ and weak acidity (pH = 5.5) (Figure 2g, reaction 4). The classical Fenton‐like reaction was conducted to explore possible reaction mechanism. Ag nanoparticles reacted with H_2_O_2_ to produce ·OH through the Fenton‐like reaction (Figure 2h).^[^ ^15^ ^]^ Therefore, we speculated that in response to X‐ray irradiation, Au/Ag NRs combined with H_2_O_2_ and H^+^ would accelerate the generation of ·OH and enhance Ag^+^ release (Figure 2i).

### 2.3 Au/Ag NRs for RIT Enhancement In Vitro

**Figure 3:**
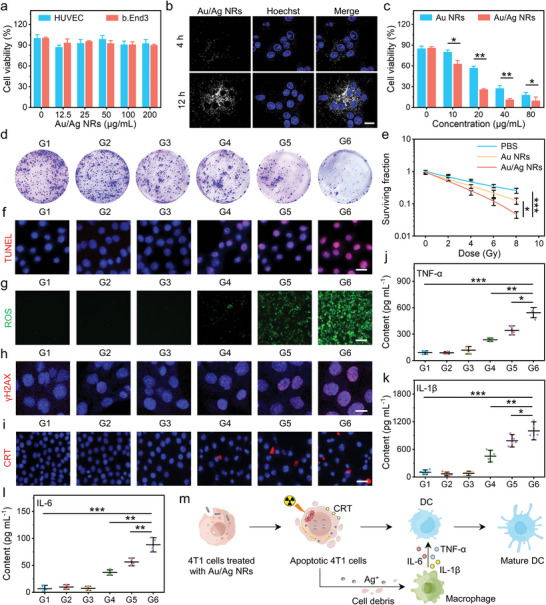
Au/Ag nanorods (NRs) for radioimmunotherapy (RIT) enhancement in vitro. a) The cytotoxicity of Au/Ag NRs against HUVECs or b.End3 cells after incubation for 24 h. b) Confocal reflection microscopy images of 4T1 cells incubated with Au/Ag NRs for 4 or 12 h. Blue: Hoechst stain; white: Au/Ag NRs; *C*_Au/Ag NRs_: 20 µg mL^−1^; scale bar: 20 µm. c) The cytotoxicity of Au NRs and Au/Ag NRs in 4T1 cells with X‐ray after 24 h of incubation. X‐ray dose: 8 Gy. d) Representative colonies of 4T1 cells of different groups. G1: PBS; G2: Au NRs; G3: Au/Ag NRs; G4: X‐ray; G5: Au NRs + X‐ray; G6: Au/Ag NRs + X‐ray. X‐ray dose: 8 Gy. e) Clonogenic survival curves of 4T1 cells with different treatments at various doses. *C*_Au NRs_: 50 µg mL^−1^; *C*_Au/Ag NRs_: 50 µg mL^−1^. f–i) Fluorescence microscopy images of 4T1 cells of different groups. TUNEL group: blue for nuclei and red for disrupted nuclei (scale bar: 25 µm); reactive oxygen species (ROS) group: green for intracellular ROS production (scale bar: 120 µm); *γ*‐H2AX group: blue for nuclei and red for DNA damage (scale bar: 10 µm); calreticulin (CRT) group: blue for nuclei and red for cell surface CRT expression (scale bar: 40 µm) (*C*_Au/Ag NRs_: 50 µg mL^−1^; X‐ray dose: 8 Gy). Quantification of j) tumor necrosis factor (TNF) *α*, k) interleukin 1*β* (IL‐1*β*), and l) interleukin 6 (IL‐6) secreted by RAW 264.7 macrophages treated in G1–G6, as measured by enzyme‐linked immunosorbent assay (ELISA). m) Schematic of the cellular mechanism of RIT enhancement by Au/Ag NRs. The picture was drawn by Figdraw. ^*^ *p* < 0.05, ^**^ *p* < 0.01, ^***^ *p* < 0.001.

Before evaluating Au/Ag NRs for RIT enhancement in vitro, we first measured their cytotoxicity. After incubating Au/Ag NRs with HUVECs and b.End3 cells for 24 h, cell viability was greater than 90% (**Figure** **3**a), indicating the low cytotoxicity of Au/Ag NRs. Next, these nanomaterials were incubated with mouse‐derived erythrocytes for 12 h. The hemolysis rate was less than 0.5% (Figure S8, Supporting Information), revealing their good blood compatibility. We then investigated the uptake of Au/Ag NRs in cells by using confocal reflection microscopy (Figure 3b). A weak signal was observed in 4T1 cells after 4 h of incubation. However, strong light scattering signals appeared in the cytoplasm when the incubation time was prolonged to 12 h. This revealed that Au/Ag NRs uptake by the cells was time‐dependent (Figure S9, Supporting Information). After endocytosis, 4T1 cells were irradiated with X‐ray of 8 Gy, and the CCK‐8 results revealed the cell viability of the Au/Ag NR‐treated group was lower than that of the Au NR‐treated group (Figure 3c, Figure S10, Supporting Information). Our results show that clonogenic survival in the Au/Ag NR‐treated group was lower than in the PBS and the Au NR‐treated groups with an increase in the irradiation dose from 0 to 8 Gy (Figure 3d,e). It was demonstrated that Au/Ag NRs have high radiosensitization efficacy for killing cancer cells compared with RT alone or Au NR‐sensitized RIT.

We also investigated the effects of Au/Ag NR‐sensitized RIT on cell apoptosis, intracellular reactive oxygen species (ROS) production, DNA double‐stranded breaks, and immune response activation. Fluorescence microscopy images revealed that significant apoptosis (Figure 3f, Figure S11a, Supporting Information), high levels of intracellular ROS (Figure 3g, Figure S11b, Supporting Information), and significant DNA double‐stranded breaks (Figure 3h, Figure S11c, Supporting Information) were detected in the Au/Ag NR‐sensitized RIT group (G6) in comparison with the control groups (G1: PBS, G2: Au NRs, G3: Au/Ag NRs), the RT alone group (G4), and the Au NR‐sensitized RIT group (G5). A large amount of cellular ROS production may induce the ICD effect and enhance the immune response.^[^ ^16^ ^]^ Therefore, we examined the expression of an important ICD marker, calreticulin (CRT),^[^ ^17^ ^]^ on the membrane surface of 4T1 cells by immunofluorescence staining. CRT expression significantly increased in the G6 group compared with the G1–G5 group in confocal fluorescence imaging (Figure 3i). Ag^+^ released from Au/Ag NRs under X‐ray irradiation may also stimulate macrophages and increase inflammatory cytokine secretion, which promotes DC maturation and immunotherapy enhancement.^[^ ^18^ ^]^ To verify our speculation, 4T1 cells in the G1‐G6 groups were incubated with macrophages (RAW 264.7 cells) for 24 h and the concentrations of interleukin 6 (IL‐6), tumor necrosis factor *α* (TNF‐*α*), and IL‐1*β*, and in the cell medium were obtained by the enzyme‐linked immunosorbent assay (ELISA) (Figure 3j–l). It was shown that cytokine expression in group G6 was higher than in groups G1‐G5. Therefore, we speculated that Au/Ag NRs not only enhance the efficiency of X‐ray RIT by producing high levels of ROS, but that they also release Ag^+^ to promote cytokine secretion from macrophages and DC maturation (Figure 3m).

### 2.4 Au/Ag NRs for X‐Ray Activated NIR‐II PA Imaging

**Figure 4:**
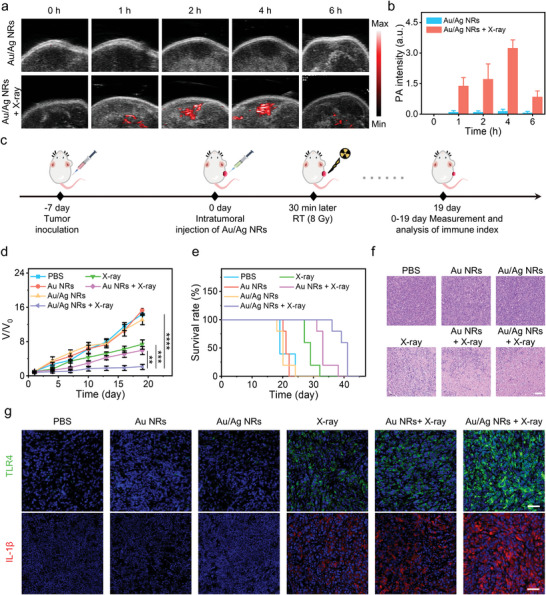
Au/Ag nanorods (NRs) for X‐ray activated second near‐infrared region (NIR‐II) photoacoustic (PA) imaging and radioimmunotherapy (RIT) in vivo. a) PA imaging of Au/Ag NRs injected into tumors intratumorally at different time points without or with X‐ray of 8 Gy. *C*_Au/Ag NRs_: 200 µg mL^−1^. b) Quantification of the NIR‐II PA signal of (a). c) Schematic of Au/Ag NRs for RIT in vivo. d) In vivo tumor growth inhibition curves and e) survival curves of mice. f) Representative images of hematoxylin and eosin (H&E) staining of tumor sections. Scale bar: 50 µm. g) Immunofluorescence images of tumor sections. Blue: DAPI staining; green: TLR4‐Alexa Fluor 488 staining; red: IL‐1*β*‐Cy3 staining; scale bar: 25 µm. *p* < 0.05, *p* < 0.01, *p* < 0.001, *p* < 0.0001.

The excellent in vitro X‐ray activated performance of Au/Ag NRs encouraged us to investigate their NIR‐II PA imaging potential in vivo. The Au/Ag NRs were injected intratumorally into the tumor region in 4T1 tumor‐bearing mice and then irradiated using high‐energy X‐ray (X‐ray dose: 8 Gy). The NIR‐II PA signal of the tumor region was monitored over time using a commercialized ultrasound/PA dual‐modality imaging system (**Figure** **4**a). The Au/Ag NR + X‐ray group exhibited a NIR‐II PA signal that was 22.6‐times higher than in the Au/Ag NR group after 4 h of X‐ray irradiation and the signal‐to‐background ratio (SBR) was measured to be 24.4 (Figure 4b). This suggested that high‐energy X‐ray effectively activated Au/Ag NRs to produce strong NIR‐II PA signals in tumor tissues, which could be used to visualize the release of Ag^+^ and to assess the therapeutic response to RIT.

### 2.5 Au/Ag NRs for Tumor RIT Enhancement in Primary Tumor‐Bearing Mice

Based on the excellent antitumor efficacy in vitro and the X‐ray activated NIR‐II PA imaging ability in vivo of Au/Ag NRs, we investigated their antitumor efficiency in 4T1 tumor‐bearing female BALB/c mice. The detailed therapeutic process is shown in Figure 4c. We classified the mice into six groups randomly (*n* = 5 mice): (i) PBS, (ii) Au NRs, (iii) Au/Ag NRs, (iv) X‐ray, (v) Au NRs + X‐ray, (vi) Au/Ag NRs + X‐ray. Compared with groups i‐v, tumor growth of group vi was the slowest (Figure 4d), indicating that Au/Ag NRs had an excellent ability to enhance RIT. Furthermore, 4T1 tumor‐bearing mice in group vi survived 42 days, which was longer than that of groups i‐v (i: 24 days, ii: 22 days, iii: 24 days, iv: 32 days, v: 38 days) (Figure 4e), indicating that Au/Ag NRs act as radiosensitizers with high antitumor efficacy. Hematoxylin and eosin (H&E) staining tumor sections of group vi showed pyknotic or absent nucleus (Figure 4f). Meanwhile, tumor cell proliferation was significantly inhibited in group vi (Figure S12, Supporting Information). Immunofluorescence staining of tumor tissues showed high concentrations of inflammatory cytokines (TLR4 and IL‐1*β*) in the tumors treated with Au/Ag NRs under X‐ray irradiation (Figure 4g). These cytokines recruit other types of immune cells, such as cytotoxic T lymphocytes (CTLs), for antitumor immunotherapy.^[^ ^19^ ^]^ The abundance of cytokines indicated that the antitumor immune response had been effectively activated. Additionally, no significant change in the body weight of mice during the treatment period indicated that the side effects were minimal (Figure S13, Supporting Information). Therefore, RIT enhancement using Au/Ag NRs increased the antitumor efficacy.

### 2.6 Au/Ag NRs for RIT Enhancement in Metastatic Tumor‐Bearing Mice

**Figure 5:**
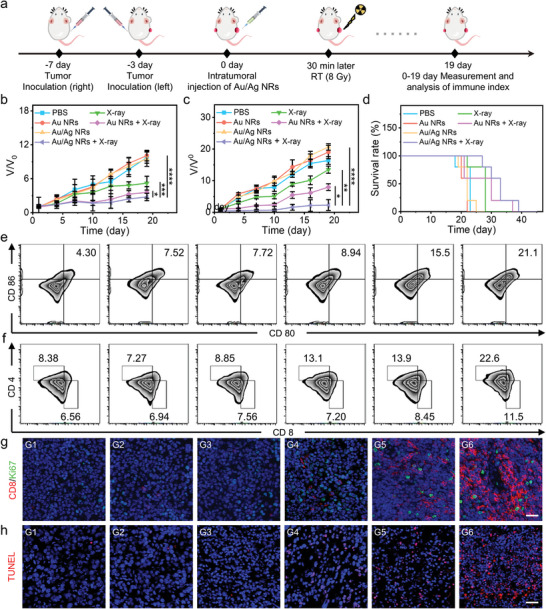
Au/Ag nanorods (NRs) for in vivo radioimmunotherapy (RIT) in metastatic tumor‐bearing mice. a) Illustration of the treatment regimen used in the distant metastasis tumor. b) Primary tumor growth curves. c) Distant tumor growth curves. d) Mice survival curves. e) Flow cytometry analysis of CD80*^+^*/CD86*^+^* dendritic cell (DC) maturation. f) Flow cytometry analysis of CD8*^+^* and CD4*^+^* T‐cell populations after treatment in distal tumor tissues. g) Representative images of CD8/Ki67 immunofluorescence staining of spleen tissues in response to different treatments. Blue: DAPI staining; red: CD8‐Cy3 staining; green: Ki67‐Alexa Fluor 488 staining; scale bar: 25 µm. h) Representative TUNEL immunofluorescence images of distant metastasis tumor sections in the different treatment groups. Blue: DAPI staining; red: TUNEL‐Cy3 staining; scale bar: 25 µm. (G1: PBS; G2: Au NRs; G3: Au/Ag NRs; G4: X‐ray; G5: Au NRs + X‐ray; G6: Au/Ag NRs + X‐ray). ^*^ *p* < 0.05, ^**^ *p* < 0.01, ^***^ *p* < 0.001, ^****^ *p* < 0.0001.

Next, 4T1 bilateral tumor mouse models were constructed to mimic distant metastatic tumors to investigate the anticancer performance of Au/Ag NR‐sensitized RIT. The right tumor tissue of mice was irradiated with X‐ray (8 Gy) 30 min after local injection of 50 µL Au/Ag NRs (200 µg mL^−1^) (**Figure** **5**a). No significant change in the body weight of mice during the treatment (Figure S14, Supporting Information). The growth curves of both the right and left tumors were recorded over the next 19 days. As shown in Figure 5b, the tumor growth on the right side was significantly inhibited, which is consistent with the results obtained in the primary mouse models. It is noteworthy that the tumor growth on the left side was inhibited along with the shrinkage of the primary tumor (Figure 5c). Left‐sided tumor growth in the Au/Ag NR + X‐ray group was slower than in the PBS, Au NR, Au/Ag NR, X‐ray, and Au NR + X‐ray groups. This observation revealed that the Au/Ag NR + X‐ray group experienced immune effects that led to the inhibition of distant tumor growth. To explore its immunotherapeutic mechanism, the number of mature DCs in the tumor‐draining lymph nodes was measured by flow cytometry. The number of CD80^+^/CD86^+^ DCs in the Au/Ag NR + X‐ray group was 4.9‐times higher than that in the control group (Figure 5e; Figure S15a, Supporting Information). It suggests that dual stimulation of local RT and Ag^+^ release promoted DC maturation. Next, we investigated the effects of DC maturation on the activation of T lymphocytes in distal tumor tissues. To this end, the quantification of CD4^+^ and CD8^+^ T cells in the left‐sided tumor tissue was analyzed by flow cytometry (Figure 5f). The proportion of activated T helper cells (CD4^+^) and cytotoxic T cells (CD8^+^) in the Au/Ag NR + X‐ray group was 14.22% and 4.94% higher, respectively, than in the PBS control group. This revealed that Au/Ag NR‐sensitized RT combined with Ag^+^ release promotes T‐cell activation and infiltration in the distal tumor region (Figure S15b,c, Supporting Information). Finally, immunofluorescence staining of interferon‐*γ* (IFN‐*γ*)/CD8 in these tumor tissues showed that the intensity of red (IFN‐*γ* ^+^) and green (CD8^+^) fluorescence signals in the Au/Ag NR + X‐ray group was greater than in the PBS control group, demonstrating that activated antitumor IFN‐*γ* ^+^/CD8^+^ T cells in the Au/Ag NR + X‐ray group were more than in the PBS control group (Figure S16, Supporting Information). We also found that the number of CD8^+^/Ki67^+^ T cells in the spleen tissue of the Au/Ag NR + X‐ray group was higher than the other five groups. These results verified that the activated T cells had a strong proliferative ability and maintained their high tumor‐killing activity (Figure 5g). The number of apoptotic cells in the Au/Ag NR + X‐ray group was higher than in the X‐ray and Au NR + X‐ray groups (Figure 5h). Mice in the Au/Ag NR + X‐ray group survived 39 days, which was longer than the PBS (23 days), Au NR (22 days), and Au/Ag NR (25 days) groups. The X‐ray (28 days) and Au NR + X‐ray (37 days) groups showed good RIT performance enhancement (Figure 5d).

### 2.7 Side Effects of Tumor RIT Enhanced by Au/Ag NRs

**Figure 6:**
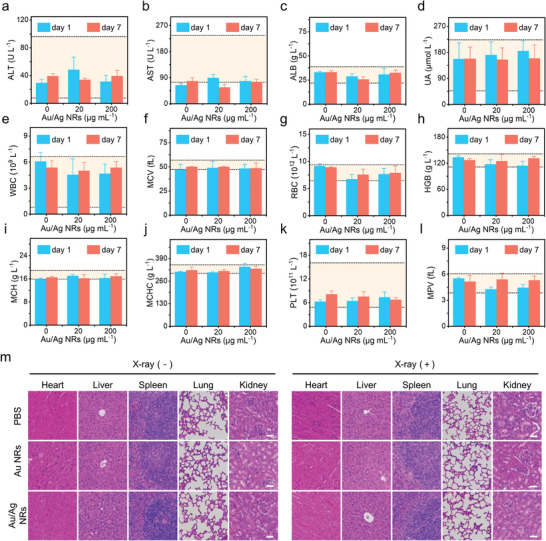
Biosafety assessment of Au/Ag nanorods (NRs) in vivo. a–l) Hematologic indices and blood biochemistry of mice that were intratumorally injected with Au/Ag NRs on days 1 and 7. The area marked by the dotted line (light yellow) was the reference value of the standard. (ALT, alanine aminotransferase; AST, aspartate aminotransferase; ALB, albumin; UA, uric acid; WBC, white blood cell; MCV, mean corpuscular volume; RBC, red blood cell; HGB, hemoglobin; MCH, mean corpuscular hemoglobin; MCHC, mean corpuscular hemoglobin concentration; PLT, platelet; MPV, mean platelet volume). m) Hematoxylin and eosin (H&E) staining of major organs sections after exposure to various treatments. Scale bar: 50 µm.

The side effects of Au/Ag NR‐sensitized RIT were evaluated 19 days after treatment. We collected blood from the mice for blood routine and biochemical analyses. The indices of the Au/Ag NR + X‐ray group were within the normal limits in comparison with healthy mice and the reference value of the standard (**Figure** **6**a–l). No inflammation, hemorrhage, or necrosis was observed in tissue sections with H&E staining of major organs (Figure 6m). Results as shown above together suggest that the side effects of Au/Ag NR‐sensitized RIT were negligible.

## 3 Conclusion

In conclusion, we successfully prepared X‐ray activatable Au/Ag core/shell NRs for tumor RIT enhancement, while achieving NIR‐II PA imaging to monitor the therapeutic response. Our results show that H_2_O_2_ (5 × 10^−3^ m) and weak acidity (pH = 5.5) accelerated Ag^+^ release during X‐ray etching of Au/Ag NRs and boosted the activatable response of NIR‐II PA imaging. Au/Ag NRs enhanced the performance of RT by producing high levels of ROS to trigger effective ICD while releasing Ag^+^ to stimulate macrophages, in turn increasing the secretion of inflammatory cytokines, promoting DC maturation, and enhancing immunotherapy. Ag^+^ release enhanced the PA imaging of Au/Ag NRs in the NIR‐II region, providing therapeutic feedback. RIT based on Au/Ag NRs achieved good antitumor effects in primary and metastatic tumor‐bearing mice. Based on RIT, the survival of metastatic mice was extended by 16 days compared with control mice, and no side effects were detected during treatment. The all‐in‐one Au/Ag NR sensitizer achieved tumor RIT enhancement and allowed for real‐time monitoring of the treatment process through NIR‐II PA imaging.

## 4 Experimental Section

### Materials

Sodium borohydride (NaBH_4_, 99%), tetrachloroauric acid (HAuCl_4_·3H_2_O, 99.99%), silver nitrate (AgNO_3_, 99.8%), hydroquinone (99%). Polyvinylpyrrolidone (molecular weight 40 000) was obtained from Sigma–Aldrich. Aladdin supplied cetyltrimethylammonium bromide (CTAB, 99%) and ascorbic acid (AA, 99.9%). Methoxypolyethylene glycol thiol (mPEG‐SH, molecular weight 2000) was purchased from Xian RuiXi Biotech. Beyotime Biotechnology provided the Cell Counting Kit‐8. Deionized water was the solvent for all solutions.

### Synthesis of Au NRs

Au NRs were obtained by the seedless approach according to the method outlined previously.^[^ ^20^ ^]^ The typical preparation process was as follows: HAuCl_4_ solution (10 × 10^−3^ m, 0.4 mL) and AgNO_3_ solution (100 × 10^−3^ m, 22.5 µL) were added to the CTAB solution (0.1 m, 10 mL) under gentle stirring to obtain the growth solution. Next, hydrochloric acid (1 m, 40 µL) and hydroquinone aqueous solution (0.1 m, 525 µL) were added to the growth solution and stirred continuously. Fifteen minutes later, the orange growth solution became clarified. Finally, the growth solution was added with 50 µL newly prepared cold NaBH_4_ (0.01 m, aq), and Au NRs were obtained after standing at room temperature overnight.

### Preparation of PEGylated Au/Ag NRs

Au/Ag NRs were obtained based on previous work.^[^ ^21^ ^]^ Five milliliters of 1 wt% polyvinylpyrrolidone was mixed with Au NR solution (1 mL), then AA (0.125 mL, 0.1 m) and AgNO_3_ (1.5 mL, 1 × 10^−3^ m) were added while stirring. The mixture changed to green after the addition of sodium hydroxide solution (0.1 m, 250 µL). The sediment was dissolved in deionized water after centrifugation (10 000 rpm, 30 min) of the solution, and the CTAB‐Au/Ag NR solution was obtained. Under violent stirring, an equal volume of mPEG‐SH solution (0.2 × 10^−3^ m) was mixed with the CTAB‐Au/Ag NR dispersion. The mixture was treated with ultrasound for 5 min and stirred for 2 h. Next, the excess mPEG‐SH was removed by centrifugation (12 000 rpm, 30 min) of the solution and abandonment of the supernatant. Finally, the sediment was redispersed in deionized water to obtain the PEGylated Au/Ag NRs.

### Characterization

TEM, energy dispersive X‐ray spectroscopy, and elemental mapping were obtained by the JEM2100F microscope (JEOL, Japan). Inductively coupled plasma optical emission spectrometry (ICP–OES) (Optima 7000DV, PerkinElmer, USA) was used to detect the Au and Ag content. A small animal irradiator (RS2000pro‐225, USA) was used to irradiate nanomaterials. The absorption band of nanomaterials was measured using an ultraviolet‐visible‐NIR spectrophotometer (UV‐2600, Shimadzu, Japan). The Malvern Zeta Sizer (Malvern, NanoZS, UK) was used for the measurement of the size and zeta potential of the NRs.

### Release of Ag^+^

Release of Ag^+^ with high‐energy X‐ray irradiation was determined as follows: 1 mL Au/Ag NR (100 × 10^−6^ m, aq) was irradiated (0, 2, 4, 6, and 8 Gy). The obtained sample was filtered by centrifugation (4000 *g* for 30 min) in 3‐kDa ultrafiltration tubes, and the filtrate was collected to measure Ag^+^ by ICP–OES. Meanwhile, the precipitate was dissolved in deionized water and used for TEM imaging.

### ESR Spectra Measurements

The ESR spectra of different chemical reactions were measured using an ESR spectrometer (Bruker EMXnano) to determine the generation of ·OH by DMPO under different conditions. Fifty microliters of the treated samples was injected into glass capillary tubes and placed in the ESR cavity, and after 2 min, the ESR spectra were documented.

### X‐Ray Activatable Performance of Au/Ag NRs

The prepared Au/Ag NRs were treated as follows: 1. Au/Ag NRs + X‐ray; 2. Au/Ag NRs + H_2_O_2_ (pH 7.4); 3. Au/Ag NRs + H_2_O_2_ (pH 5.5); 4. Au/Ag NRs + X‐ray + H_2_O_2_ (pH 5.5), H_2_O_2_: 5 × 10^−3^ m, 1 mL; Au/Ag NRs: 50 µg mL^−1^, 1 mL; X‐ray: 8 Gy. Then, the UV absorption wavelengths of the solutions of different reaction groups were detected at different time points.

X‐ray activatable properties of the Au/Ag NR solution were determined by the NIR‐II PA imaging system (Vevo LAZR‐X, Fujifilm VisualSonics, USA). Four groups (*n* = 3) samples were analyzed: (i) Au/Ag NRs + 8 Gy X‐ray; (ii) Au/Ag NRs + H_2_O_2_ (5 × 10^−3^ m, pH = 7.4); (iii) Au/Ag NRs + H_2_O_2_ (5 × 10^−3^ m, pH = 5.5); (iv) Au/Ag NRs + H_2_O_2_ (5 × 10^−3^ m, pH = 5.5) + 8 Gy X‐ray. These solutions were placed in a transparent tube and subjected to NIR‐II PA imaging (laser wavelength: 1064 nm) after 0, 1, 2, and 3 h of the reaction.

For in vivo imaging, BALB‐c/nude tumor‐bearing mice were randomly divided into two groups (*n* = 3). Following injection of 50 µL Au/Ag NR solution (200 µg mL^−1^) intratumorally, the tumor region was subjected to X‐ray irradiation of 8 Gy. NIR‐II PA imaging was performed after irradiation for 0, 1, 2, 4, and 6 h. VolView software was used for PA image processing, and quantitative analysis of the PA signals was performed using Image J software.

### In Vitro Cytotoxicity

HUVECs and b.End3 cells, which were used to represent normal cells, were inoculated in 96‐well plates (8 × 10^3^ cells per well) and cultured for 24 h under 37 °C. Then, 100 µL medium containing Au/Ag NRs (0–80 µg mL^−1^) was added to each well. 24 h later, the samples were irradiated with X‐ray (8 Gy) and cultured under 37 °C for a further 24 h. Following adding 10 µL of CCK‐8, microplate readers (PerkinElmer) were used to determine the absorbance at 450 nm of each well (*n* = 5 per well) before calculating cell survival.

Next, the hemolysis of the Au/Ag NRs was evaluated. Blood was collected from BALB/c mice and centrifuged (2000 rpm, 3 min). The blood was washed three times with PBS until the supernatant was not red in color. Then, erythrocytes were prepared into a 2% suspension with saline. Five hundred microliters suspension was incubated at 37 °C with 500 µL Au/Ag NRs (7.8–500 µg mL^−1^) at different concentrations for 2 h. The absorbance of the supernatant was determined by employing a microplate reader (PerkinElmer) to calculate the percentage of hemolysis. Positive and negative controls were water and PBS, respectively.

### Cellular Uptake Assay

The confocal reflection microscopy with a light‐scattering imaging module was used to assess the ability of 4T1 cells to take up the Au/Ag NRs. 4T1 cells (2 × 10^4^) were inoculated in 8‐well confocal plates, cultured for 24 h, and incubated with Au/Ag NRs (20 × 10^−6^ m) for 0, 4, and 12 h, respectively. Afterward, the cells were fixed with paraformaldehyde of 4%, and stained with Hoechst of 5 µg mL^−1^ for confocal reflection microscopy.

Second, this work quantitatively measured Au/Ag NR uptake by 4T1 cells using flow cytometry. Au/Ag NRs were labeled with the fluorescent dye FITC. Next, FITC‐labeled Au/Ag NRs were incubated with the 4T1 cells (1 × 10^5^) in a 6‐well plate. After 0, 4, or 12 h, the free Au/Ag NRs were removed using centrifugation, and the cells were collected for flow cytometry analysis.

### Colony Formation Experiments

4T1 cells were inoculated in 96‐well plates (1 × 10^3^ per well). After 12 h of incubation, the six groups are as follows: (*n* = 5 per group): (i) PBS, (ii) Au NRs, (iii) Au/Ag NRs, (iv) X‐ray, (v) Au NRs + X‐ray, (vi) Au/Ag NRs + X‐ray (Au NRs = 50 µg mL^−1^, Au/Ag NRs = 50 µg mL^−1^). There were five doses of X‐ray: 0, 2, 4, 6, and 8 Gy. Six days later, paraformaldehyde (4%) was used to fix the cells and a solution of 0.4% crystal violet (w/v) in 20% methanol was used to stain the cells. The cell survival fraction was calculated after counting. Inhibition of cell proliferation after different treatments was assessed by measuring the survival fraction.

### Cell Apoptosis Detection

4T1 cells were inoculated in a 6‐well plate (1 × 10^5^ cells per well). After 24 h of incubation, cell grouping and processing were the same as in Section 4.10. Hoechst stain was applied to the fixed cells after they had been fixed in paraformaldehyde (4%). Finally, fluorescence microscopy was performed using the Nikon Ti2‐E fluorescence microscope.

### ROS Detection

4T1 cells were treated in the same way as in Section 4.10. After different treatments, the cells were incubated with DCFH‐DA (ROS probe, Biyuntian, 10 × 10^−6^ m) for 30 min. ROS imaging was performed using the Nikon Ti2‐E fluorescence microscope.

### DNA Double‐Stranded Break Detection

4T1 cells were seeded into proprietary confocal 8‐well plates (2 × 10^4^ cells per well). After 24 h of incubation, it was treated in the same process as described in Section 4.10. After 1 h, paraformaldehyde (4%) was applied to fix the cells, and then 5% bovine serum albumin was used to block the samples for 1 h at 37 °C, before being co‐incubated with anti‐*γ*‐H2AX primary antibody (1:1000 dilution) and FITC‐labeled goat anti‐mouse immunoglobulin G (1:1000 dilution), respectively, followed by addition of *γ*‐H2AX and staining with Hoechst stain (5 µg mL^−1^). Finally, laser confocal fluorescence microscopy (Olympus IX73) was performed, and the focal density of *γ*‐H2AX (foci 100 µm^−2^) was quantified with Image J software.

### Calreticulin Detection

4T1 cells were inoculated in 96‐well plates (8 × 10^3^ cells per well). After 24 h of incubation, the six groups were grouped as above (*n* = 5). After treating cells with CRT primary antibody for 1 h, they were washed three times using PBS containing 0.5% Triton X‐100. The cells were cultured with Cy3‐conjugated secondary antibody for 30 min and applied with Hoechst stain (5 µg mL^−1^). Finally, fluorescence microscopy (Nikon Ti2‐E) was performed for CRT detection.

### In Vitro Cytokine Detection

4T1 cells were inoculated in 6‐well plates (1 × 10^5^ cells per well). After 24 h of incubation, the cells were subjected to different treatments. Then the cells were cultured for another 12 h, and the supernatant was collected and incubated with RAW 264.7 macrophages for 24 h at 37 °C under 5% carbon dioxide conditions. Cytokines in the supernatant (TNF‐*α*, IL‐6, and IL‐1*β*) were quantitatively measured using ELISA. A microplate reader (PerkinElmer) was used to determine the absorbance at 450 nm. The concentrations of cytokines secreted by macrophages in the different groups were calculated using standard curves.

### Animal Experiments

Vital River (Beijing, China) provided BALB/c and BALB/c nude female mice. The Institutional Animal Care and Use Committee of Shenzhen Institute of Advanced Technology, Chinese Academy of Sciences had approved animal experiments (Ethics Approval No.: SIAT‐IACUC‐200728‐YGS‐SZH‐A1337).

A subcutaneous primary 4T1 tumor mouse model was constructed as follows: Subcutaneous injection of 100 µL 4T1 cell suspension (1 × 10^6^ cells mL^−1^) was performed in the right hind limb of each BALB/c mouse. Experimental mice bearing tumors of 50 to 80 mm^3^ were used for in vivo experiments.

A subcutaneous metastatic 4T1 tumor mouse model was constructed as follows: Subcutaneous injection of 100 µL 4T1 cell suspension (1 × 10^6^ cells mL^−1^) was performed in the right hind limb of each BALB/c mouse. Five days later, the same concentration of 4T1 cell suspension (100 µL) was subcutaneously injected into the back of the left hind limb to produce the distal metastatic tumor. Experimental mice bearing tumors of 50 to 80 mm^3^ were used for in vivo experiments.

### In Vivo Antitumor Study

RIT sensitization with Au/Ag NRs was evaluated by randomly dividing BALB/c mice with primary subcutaneous 4T1 tumors into six groups (*n* = 5 mice per group): (i) PBS, (ii) Au NRs, (iii) Au/Ag NRs, (iv) X‐ray, (v) Au NRs + X‐ray, (vi) Au/Ag NRs + X‐ray (Au NRs: 200 µg mL^−1^, Au/Ag NRs: 200 µg mL^−1^, X‐ray dose: 8 Gy). Au NR solution, Au/Ag NR solution, or PBS was intratumorally injected into the tumor tissue. Thirty minutes later, the tumor sites in groups 4–6 were subjected to X‐ray irradiation. Mice were weighed and their survival was recorded. The mice were sacrificed when the right‐sided tumor volume reached 1000 mm^3^. The size of the mouse tumor was measured every other day for 19 days, as follows: *V* = *L* × *W* ^2^/2, where *V*, *L*, and *W* are the tumor volume, length, and width, respectively. The relative volume was determined by *V*/*V*_0_, where *V*_0_ is the initial volume.

To investigate the efficacy of Au/Ag NR sensitization of RIT, BALB/c mice with metastatic subcutaneous tumors were randomly divided into six groups (*n* = 5 mice per group), as detailed above. Au NR solution, Au/Ag NR solution, or PBS was injected intratumorally into the right tumor of each tumor‐bearing mouse. Thirty minutes later, the right‐sided tumors of the mice in groups 4–6 were subjected to X‐ray irradiation. Mice were weighed and their survival was recorded. The mice were considered as deceased when the right‐sided tumor volume reached 1000 mm^3^. The size of bilateral tumors was measured and recorded every other day for 19 days.

### Histological Analysis

Following 19 days of treatment, the mice were euthanized, and the heart, liver, spleen, lung, and kidney were removed and fixed in 10% paraformaldehyde, paraffin‐embedded, and sectioned (4 µm thickness). Fluorescence imaging was performed using an inverted fluorescence microscope (Nikon Ti2‐E) after H&E staining of the slices.

The tumor‐bearing mice were divided into six groups (*n* = 3) at random and the grouping was as above. After 2 days of treatment, one mouse in each group was euthanized, the tumors were removed, fixed in 10% paraformaldehyde, paraffin‐embedded, then sectioned (4 µm thickness). The slices were stained with H&E and imaged using a fluorescence microscope (Nikon Ti2‐E).

### In Vitro Immunofluorescence Staining

After treatment, the tumor and spleen tissues were removed from the mice, sliced into sections, and incubated with primary antibodies (TLR4, IL‐1*β*, IFN‐*γ*, Ki67, and CD8) and corresponding fluorescein‐labeled secondary antibodies (TLR4‐Alexa Fluor 488, IL‐1*β*‐Cy3, IFN‐*γ*‐Cy3, Ki67‐Alexa Fluor 488, CD8‐Cy3, and CD8‐Alexa Fluor 488). Sections stained with different types of fluorescein were observed and imaged by laser confocal microscopy.

### In Vitro Flow Cytometry

Tumor tissues and lymph nodes from different groups were minced with scissors and digested with collagenase. Using a 70‐µm cell filter, the dissociated cells were filtered into a 50‐mL centrifuge tube containing 5 mL erythrocyte lysate to obtain a single‐cell suspension. Following incubation for 5 min at 37 °C, 5 mL complete DMEM was used to terminate the reaction. Centrifugation at 1200 rpm for 5 min was followed by three washings in PBS and resuspension in complete DMEM for 10 mL. A 30‐min staining with anti‐CD80‐FITC and anti‐CD86‐PE was carried out to investigate the maturation of DCs. To analyze the response of T lymphocytes to different treatments in distant tumors after DC maturation, immune cell populations were measured with anti‐CD4‐PE and anti‐CD8‐FITC staining by flow cytometry.

### Statistical Analysis

Two‐tailed Student's *t*‐test and one‐way analysis of variance were used for statistical analysis of two and multigroups, respectively. *p* value < 0.05 indicates a significant difference. Significant differences are indicated by *p* values of less than 0.05. ^*^ *p* < 0.05, ^**^ *p* < 0.01, ^***^ *p* < 0.001 (unpaired two‐tailed Student's *t*‐test).

## Conflict of Interest

The authors declare no conflict of interest.

## Author Contributions

S.Z., D.G., H.Z., Y.L., and Z.S. conceived and designed the research, interpreted the data, and wrote the manuscript. S.Z. prepared and characterized Au/Ag NRs. D.G. conducted the related experiment in vitro. Y.W. conducted NIR‐II PA imaging studies. Z.L. and Y.W. conducted animal experiments. H.Z. provided suggestions for the experiment and revised the manuscript. All authors gave approval to the final version of the manuscript.

## Supporting information

Supporting Information

## Data Availability

The data that support the findings of this study are available from the corresponding author upon reasonable request.
