# Supplementary material for: X‐Ray Activatable Au/Ag Nanorods for Tumor Radioimmunotherapy Sensitization and Monitoring of the Therapeutic Response Using NIR‐II Photoacoustic Imaging
Source: Adv Sci (Weinh). 2023 Feb 15;10(11):2206979. doi: 10.1002/advs.202206979 (PMC10104665; doi:10.1002/advs.202206979)
Supplement: Supplementary file 1 — Supporting Information [file ADVS-10-2206979-s001.pdf]

Supporting Information

X-ray-activatable Au/Ag Nanorods for Tumor Radioimmunotherapy Sensitization and Monitoring of the Therapeutic Response Using NIR-II Photoacoustic Imaging

Si Zheng, Duyang Gao, Yayun Wu, Dehong Hu, Ziyue Li, Yuenan Wang, Hairong Zheng, Yingjia Li*, Zonghai Sheng**

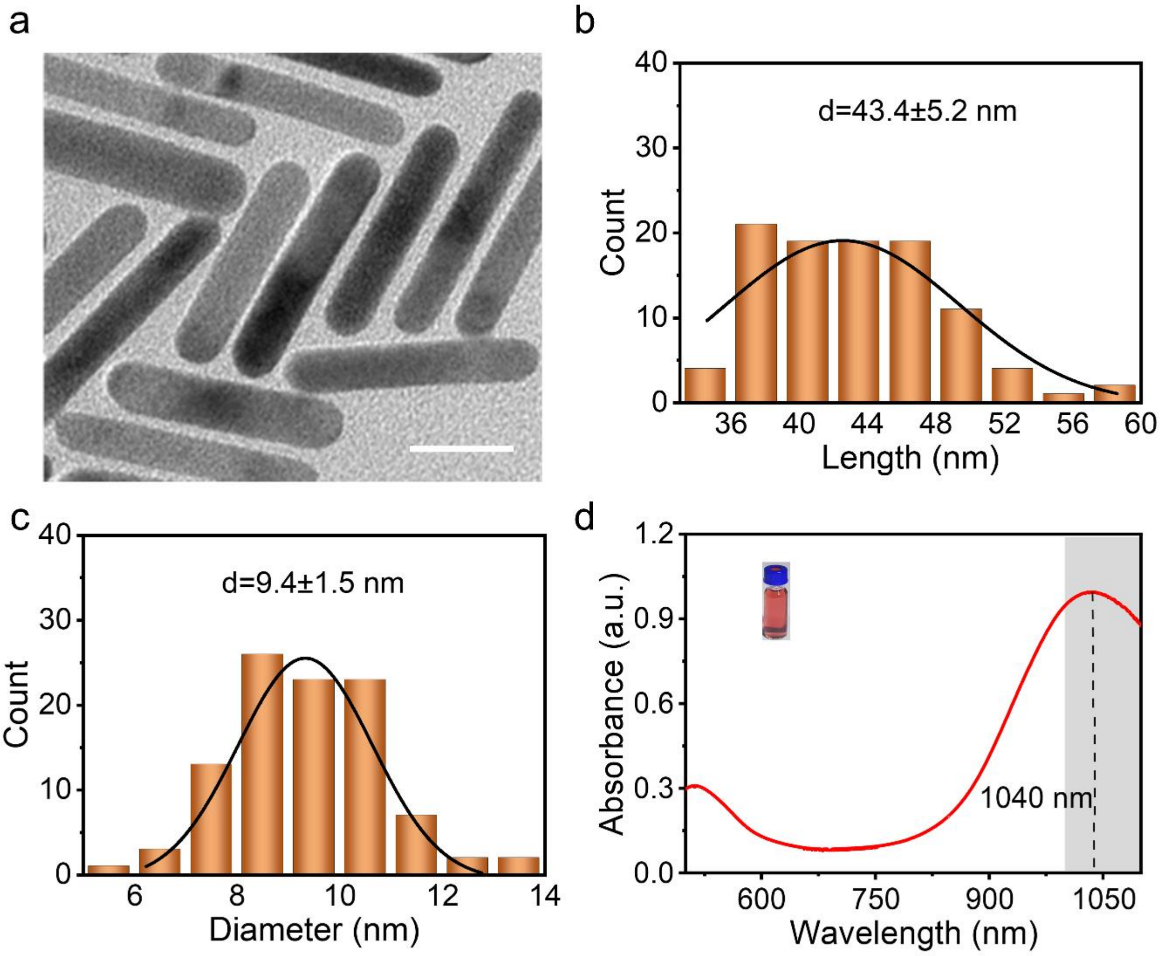

Figure S1. a) TEM image of Au NRs, scale bar: 20 nm. b-c) Length and diameter distribution of Au NRs based on TEM images. d) UV-vis absorption spectra of Au NRs. The insert is a digital photo of the Au NRs solution.

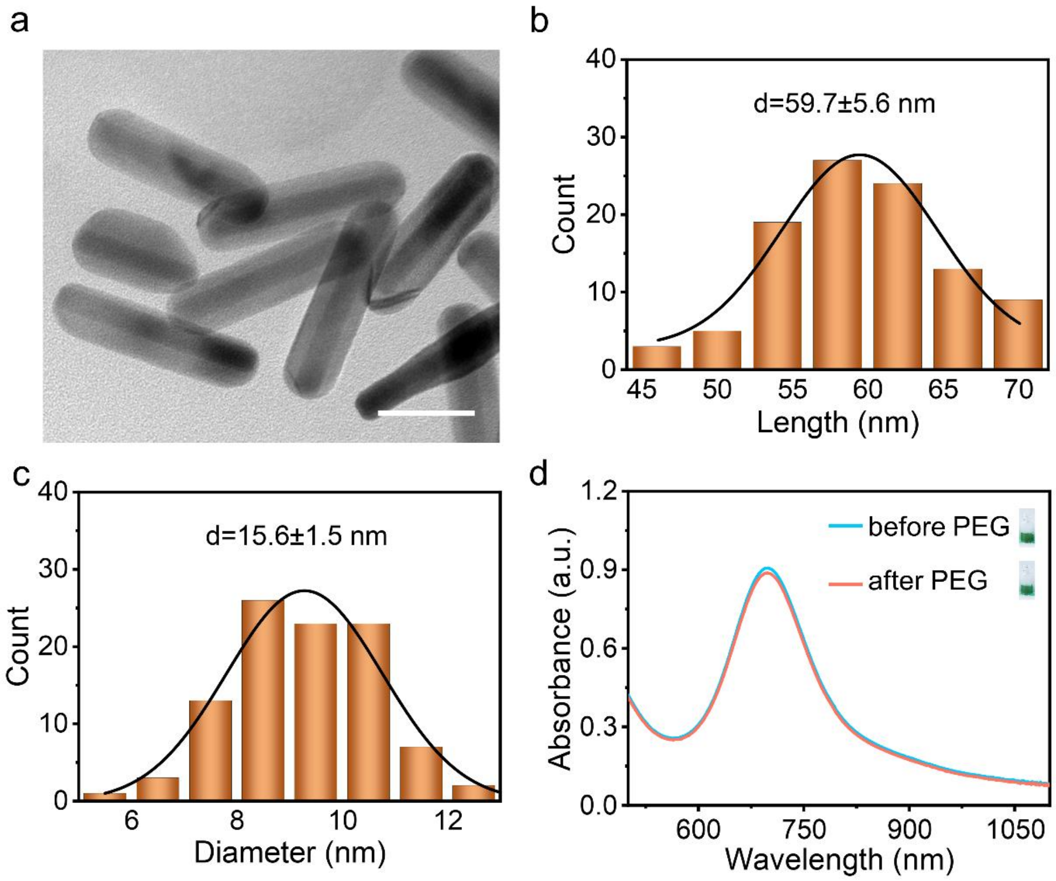

Figure S2. a) TEM image of Au/Ag NRs, scale bar: 20 nm. b-c) Length and diameter distribution of Au/Ag NRs based on TEM images. d) UV-vis absorption spectra of Au/Ag NRs and the PEGylated Au/Ag NRs. The insert is digital photos of the Au/Ag NRs and PEGlated Au/Ag NRs solution.

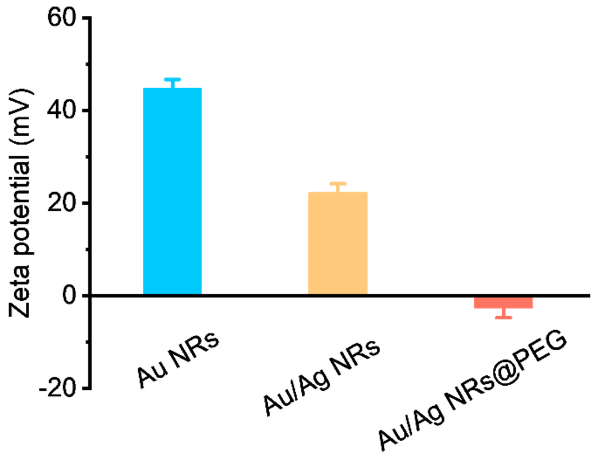

Figure S3. Zeta potential of Au NRs, Au/Ag NRs with or without PEGylation.

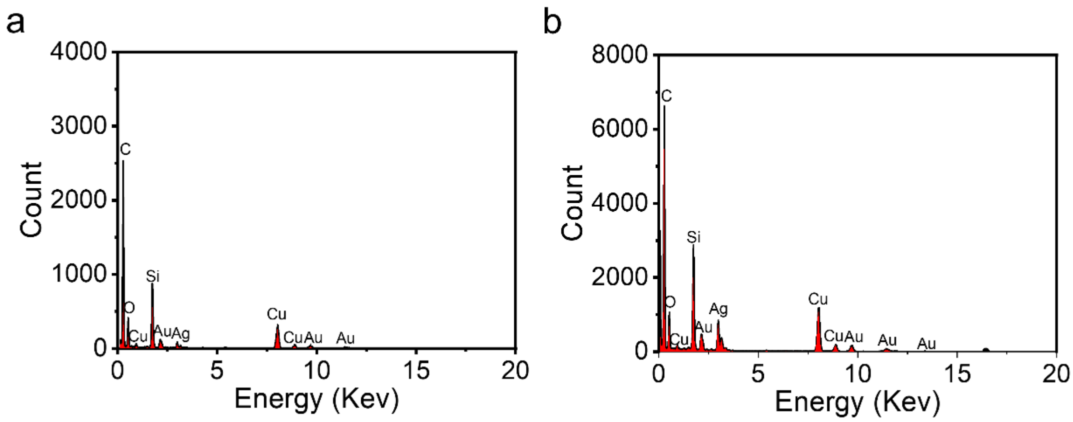

c

	Ag		Au	
	weight%	atomic%	weight%	atomic%
RT(−)	68.78	80.09	31.22	19.91
RT(+)	45.7	60.58	54.3	39.42

Figure S4. a-b) EDX spectra of Au/Ag NRs and Au/Ag NRs treated with high-energy X-ray. c) Weight and atomic of Au or Ag of Au/Ag NRs before and after X-ray treatments (dose = 8 Gy). RT (-): without X-ray irradiation, RT (+): with X-ray irradiation.

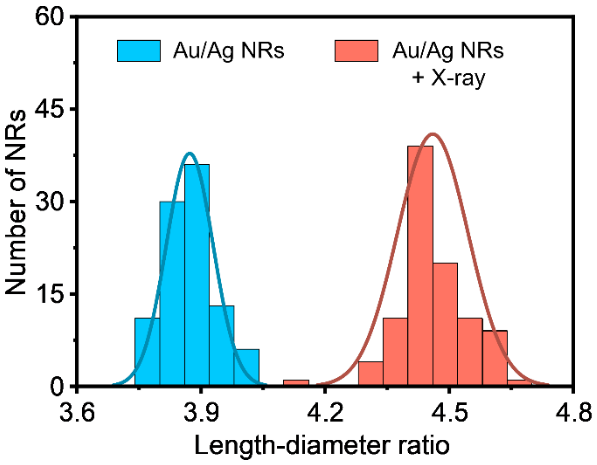

Figure S5. The distribution of length-diameter ratios of Au/Ag NRs before and after X-ray irradiation. X-ray dose: 8 Gy.

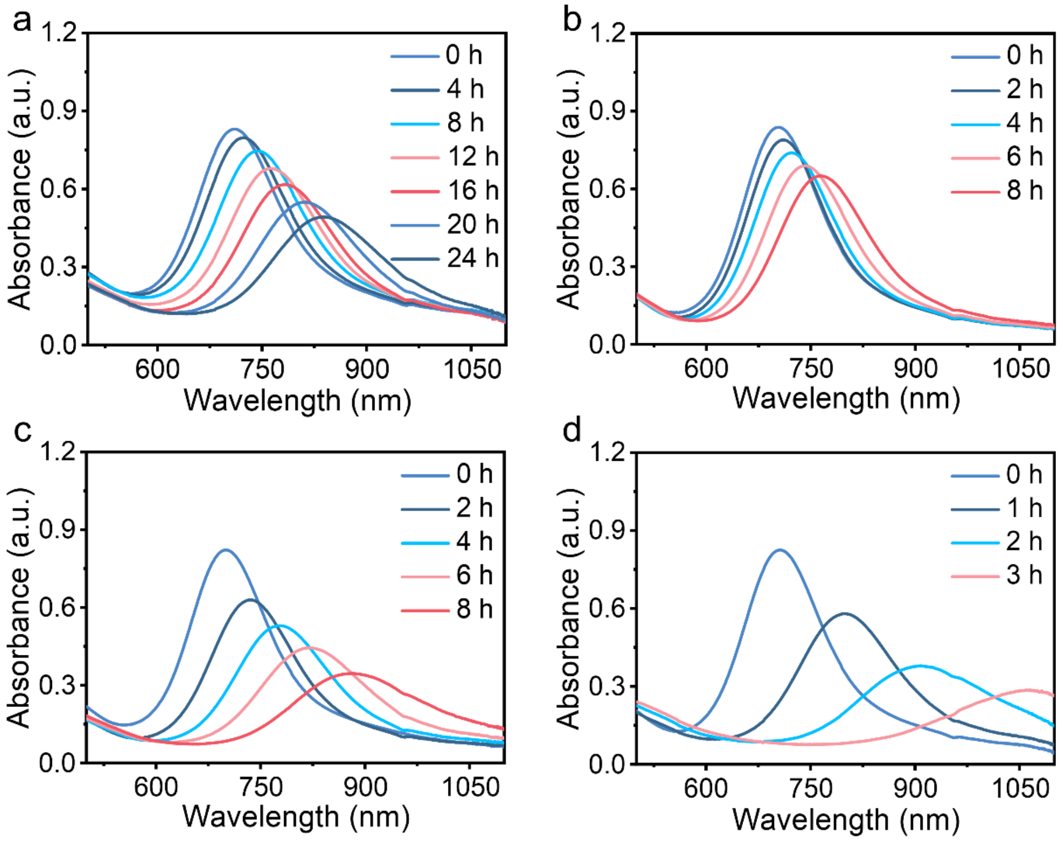

Figure S6. UV-vis absorbance spectra of Au/Ag NRs under different treatments, a) 8 Gy X-ray, b) H_2O_2 (5 mM) at pH 7.4, c) H_2O_2 (5 mM) at pH 5.5, d) 8 Gy X-ray and H_2O_2 (5 mM) at pH 5.5. $C_{Au/Ag\ NRs}$ = 50 μg mL^{-1}.

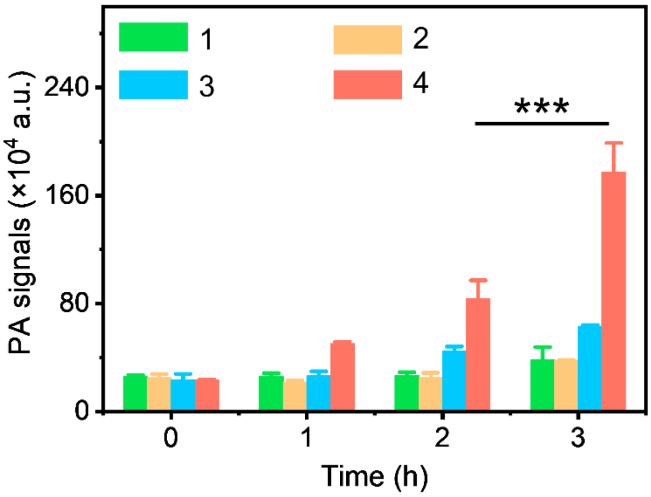

Figure S7. Quantitative analysis of NIR-II PA intensity of Au/Ag NRs in Figure 2c-2f. ***P < 0.001. 1: Au/Ag NRs + 8 Gy X-ray; 2: Au/Ag NRs + H_2O_2 (5 mM, pH = 7.4); 3: Au/Ag NRs + H_2O_2 (5 mM, pH = 5.5); 4: Au/Ag NRs + H_2O_2 (5 mM, pH = 5.5) + 8 Gy X-ray.

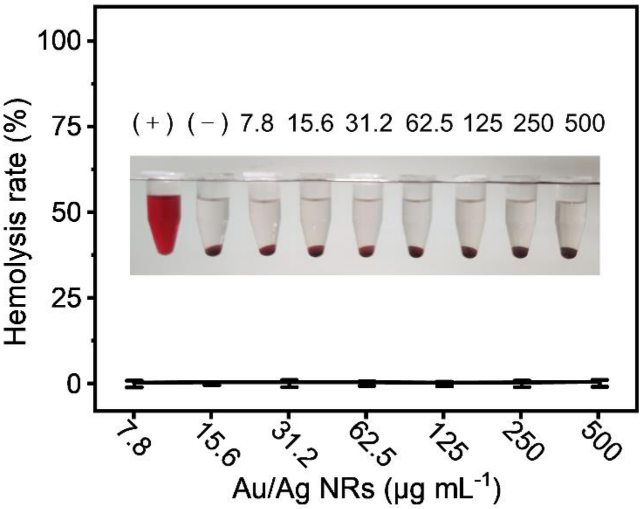

Figure S8. Hemolysis rate of Au/Ag NRs with different concentrations after incubation with red blood cells (RBCs).

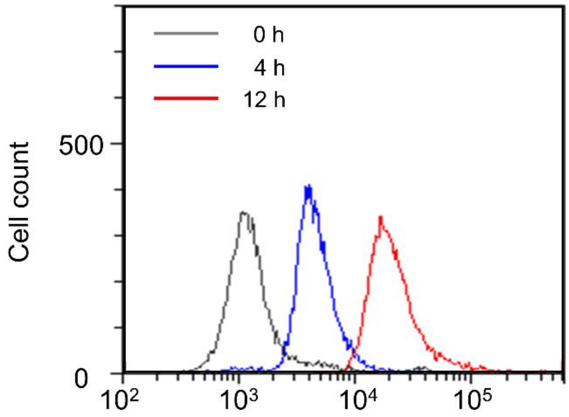

Figure S9. Flow cytometric analysis of the cellular uptake of FITC-labeled Au/Ag NRs over time.

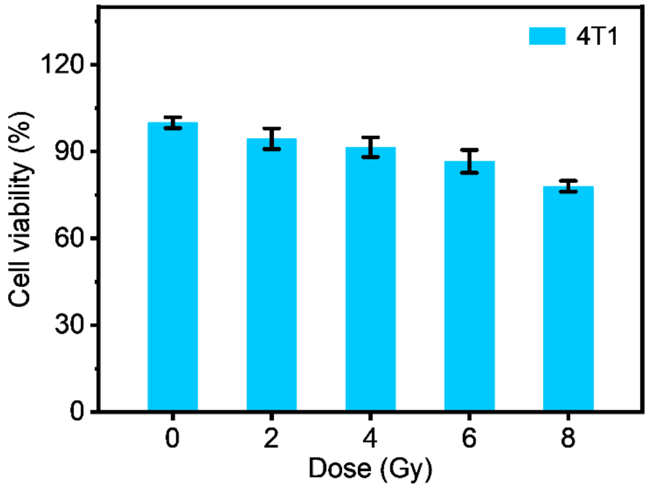

Figure S10. The cytotoxicity of 4T1 cells that irradiated by X-ray at different doses. X-ray dose: 0 Gy, 2Gy, 4Gy, 6Gy and 8Gy.

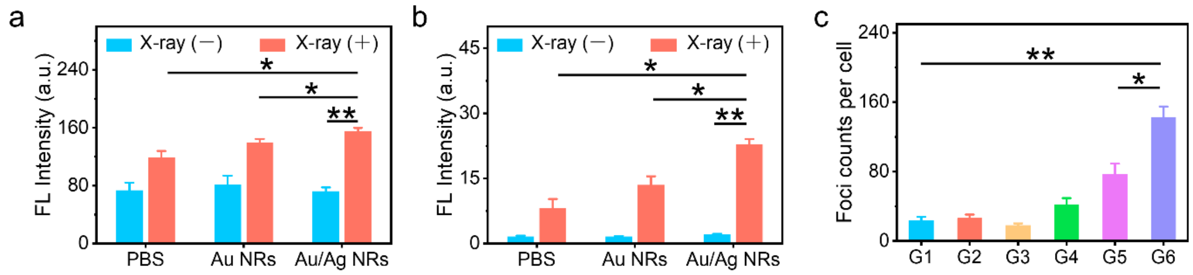

Figure S11. a) Quantitative analysis of fluorescence signal intensity in different groups in Fig. 3f and b) Fig. 3g. c) Foci counts per cell of confocal fluorescent images in Figure 3h. The data were analyzed with Image J software. G1: PBS; G2: Au NRs; G3: Au/Ag NRs; G4: X-ray; G5: Au NRs + X-ray; G6: Au/Ag NRs + X-ray. X-ray dose: 8 Gy. *$P < 0.05$, **$P < 0.01$.

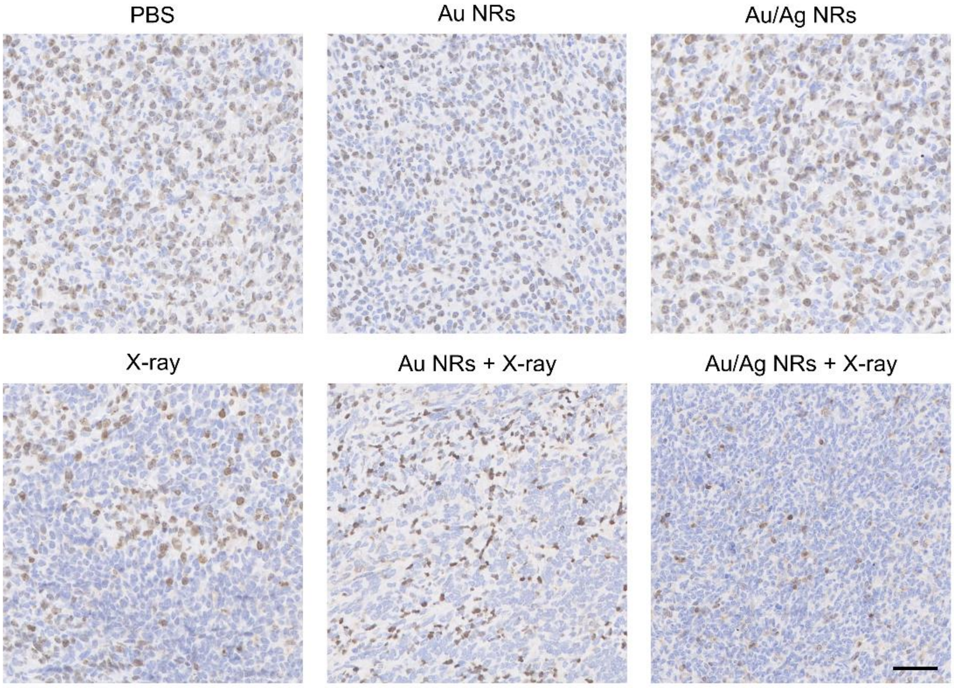

Figure S12. Representative photographs of Ki67 staining of primary tumor sections in the six groups. Scale bar: 25 μm.

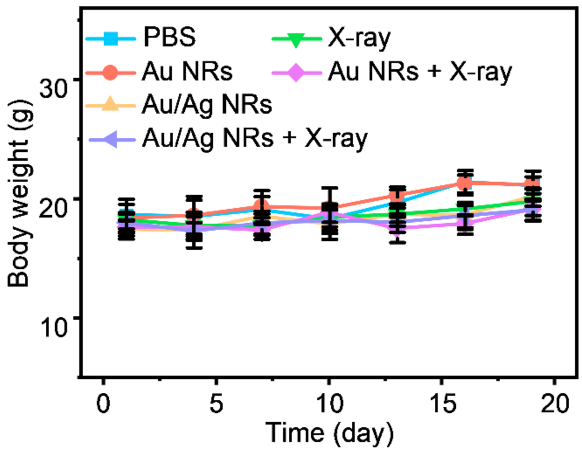

Figure S13. Body weight change curves of primary tumor-bearing mice following different treatments (n = 5).

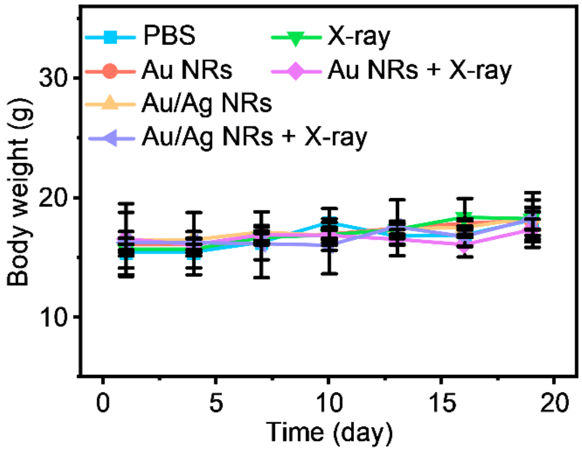

Figure S14. Body weight change curves of metastatic tumor-bearing mice following different treatments (n = 5).

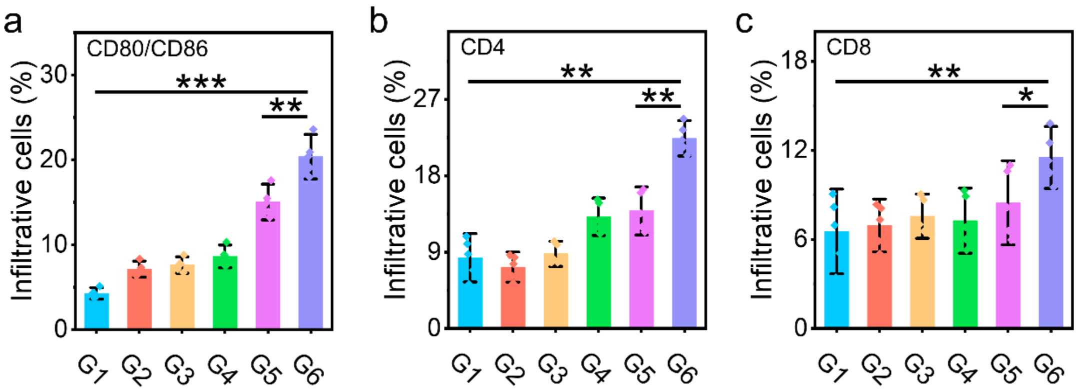

Figure S15. The quantitative analysis of a) CD80$^+$ CD86$^+$ dendritic cells, b) CD4$^+$ T cells, and c) CD8$^+$ cells. G1: PBS; G2: Au NRs; G3: Au/Ag NRs; G4: X-ray; G5: Au NRs + X-ray; G6: Au/Ag NRs + X-ray. X-ray dose: 8 Gy. *$P < 0.05$, **$P < 0.01$, ***$P < 0.001$.

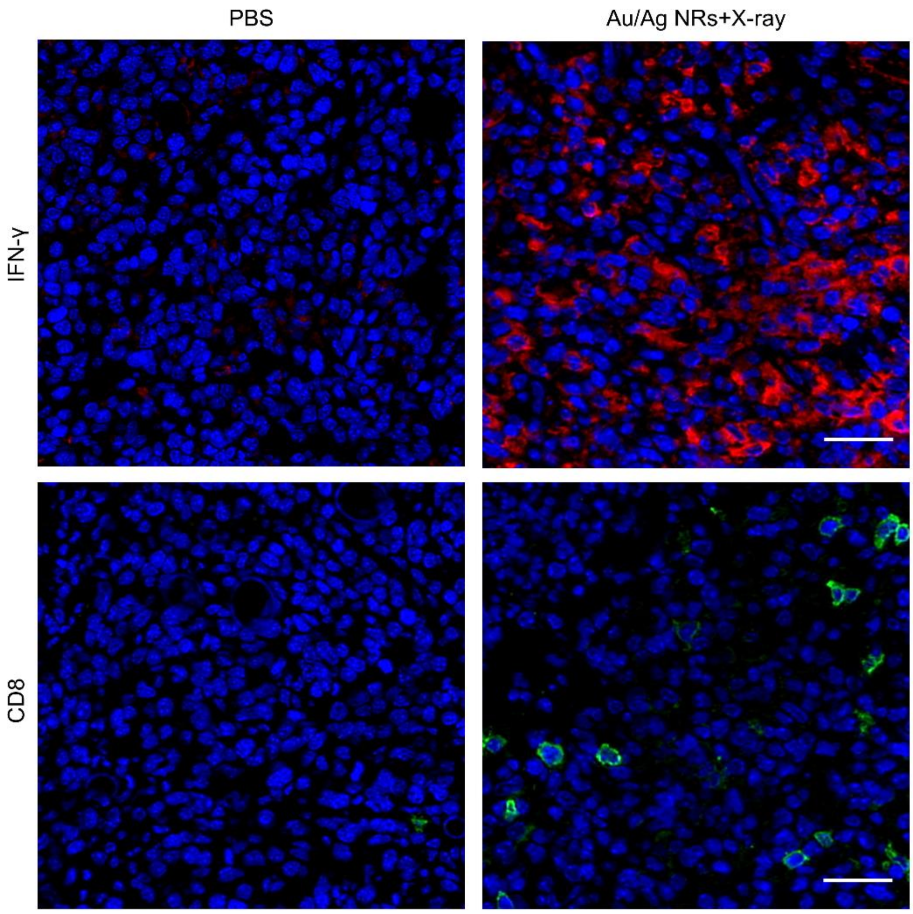

Figure S16. Immunofluorescence staining of distant tumors in group 1 (PBS) and group 6 (Au/Ag NRs + X-ray). The IFN-γ^+ (red) and $CD8^+$ (green) suggested that a powerful T cell immunotherapy occurred in tumor slices. Scale bar: 50 μm. X-ray dose: 8 Gy, $C_{Au/Ag\ NRs}$ = 200 μg mL^{-1}. Blue represents DAPI staining, red represents IFN-γ-Cy3 staining and green represents CD8-Alexa Fluor 488 staining.
